# Indirect Pathway Metabolic Engineering Strategies for Enhanced Biosynthesis of Hyaluronic Acid in Engineered *Corynebacterium glutamicum*

**DOI:** 10.3389/fbioe.2021.768490

**Published:** 2021-12-20

**Authors:** Yan Du, Fangyu Cheng, Miaomiao Wang, Chunmeng Xu, Huimin Yu

**Affiliations:** ^1^ Key Laboratory for Industrial Biocatalysis of the Ministry of Education, Department of Chemical Engineering, Tsinghua University, Beijing, China; ^2^ Center for Synthetic and Systems Biology, Tsinghua University, Beijing, China

**Keywords:** hyaluronic acid, engineered *Corynebacterium glutamicum*, IolR deletion, cardiolipin (CL), *Vitreoscilla* hemoglobin (VHb), glutamine

## Abstract

Hyaluronic acid (HA) is composed of alternating d-glucuronic acid and *N*-acetyl-d-glucosamine, with excellent biocompatibility and water retention capacity. To achieve heterologous biosynthesis of HA, *Corynebacterium glutamicum*, a safe GRAS (generally recognized as safe) host, was utilized and metabolically engineered previously. In this work, to achieve further enhancement of HA yield, four strategies were proposed and performed separately first, i.e., (1) improvement of glucose uptake via *iolR* gene knockout, releasing the inhibition of transporter IolT1/IolT2 and glucokinases; (2) intensification of cardiolipin synthesis through overexpression of genes *pgsA1*/*pgsA2*/*cls* involved in cardiolipin synthesis; (3) duly expressed *Vitreoscilla* hemoglobin in genome, enhancing HA titer coupled with more ATP and improved NAD^+^/NADH (>7.5) ratio; and (4) identification of the importance of glutamine for HA synthesis through transcriptome analyses and then enhancement of the HA titer via its supplement. After that, we combined different strategies together to further increase the HA titer. As a result, one of the optimal recombinant strains, Cg-dR-CLS, yielded 32 g/L of HA at 60 h in a fed-batch culture, which was increased by 30% compared with that of the starting strain. This high value of HA titer will enable the industrial production of HA via the engineered *C. glutamicum*.

## Introduction

Hyaluronic acid (HA), composed of alternating β-1,3-*N*-acetyl-d-glucosamine (GlcNAc) and β-1,4-d-glucuronic acid (GlcUA), belongs to the glycosaminoglycan family (Westbrook et al., 2018; Cheng et al., 2019b; Wang et al., 2020) and mainly exists in animal tissues such as chicken crowns. HA has already been applied in the clinical, cosmetic, pharmaceutical, and food industry due to its excellent biocompatibility and extraordinary water-retaining properties (Stecco et al., 2011; Graca et al., 2020; Yu et al., 2020). Generally, HAs with different molecular weights (M_W_s) have different applications (Qiu et al., 2021). High-molecular-weight HA (HMW-HA, M_W_ ≥ 1 × 10^6^ Da) is mainly used for joint cavity injection and cartilage degeneration repair, owing to its good viscoelasticity, moisture retention, and lubrication properties. Low-molecular-weight HA (LMW-HA, 1 × 10^4^–1 × 10^6^ Da) usually plays an important role in the cosmetics field (Qiu et al., 2021), since it can improve skin elasticity and reduce wrinkles (Pavicic et al., 2011), as well as regulate skin metabolism and delay aging (Camacho et al., 2016). In addition, HA oligosaccharides (M_W_ ≤ 1 × 10^4^ Da) may have significant application prospects in the food health field (Boltje et al., 2009), as they have been widely used in fruit juice, soy milk, jelly, and other food. In the future, HAs with different M_W_s will have more and better development prospects in different fields (Qiu et al., 2021).

Nowadays, industrial HA production has already been achieved through fermentation of group C *Streptococcus* (*Streptococcus equisimilis* and *Streptococcus zooepidemicus*) (Liu et al., 2011) and has gradually replaced the traditional animal issue extraction methods. However, *Streptococcus* sp. may produce some exotoxins and immunogens during HA production (Cheng et al., 2019b; Wang et al., 2020). Considering the potential hazards, adoption of GRAS (generally recognized as safe) strains to produce HA is required urgently. Up to now, HA has been biosynthesized successfully in *Escherichia coli* (Yu and Stephanopoulos 2008; Mao et al., 2009; Woo et al., 2019), *Lactococcus lactis* (Prasad et al., 2010; Sunguroglu et al., 2018; Jeeva et al., 2019), *Bacillus subtilis* (Westbrook et al., 2018a; Westbrook et al., 2018b; Li et al., 2019), *Agrobacterium* sp. (Mao and Chen 2007), and *Corynebacterium glutamicum* (Hoffmann and Altenbuchner 2014; Cheng et al., 2016; Cheng et al., 2017; Cheng et al., 2019b; Wang et al., 2020; Zheng et al., 2020). Even though the HA titers obtained from heterologous hosts are in general still lower than those achieved by natural, pathogenic producers. In the biosynthesis of HA, HA polymer is synthesized by an enzyme called HA synthase (HAS), which is grouped into two classes (Agarwal et al., 2019). Class I HAS is a kind of integral membrane protein containing a single domain, while Class II HAS is a soluble/membrane anchored protein with two domains (Weigel 2015). Most of the HAS found so far belong to Class I, such as HAS from *S. equisimilis* (seHAS) and *Streptococcus pyogenes* (spHAS); however, Class II enzyme is found only in *Pasteurella multocida* (pmHAS). It was reported that seHAS and spHAS contained a single HAS protein associated with an additional component with a mass of about 23 kDa, which was identified as cardiolipin (CL), one of the common bacterial membrane phospholipids (Weigel 2002). Thus, the active HAS enzyme contains a HAS protein monomer and about 14–18 CL molecules as a complex, in which CL is essential for enzymatic activity. It has not been studied whether enhanced CL synthesis would have an impact on HA production.

In order to meet the requirements of industrial production, researchers have made lots of efforts to increase the yield of HA in recombinant strains. Some hosts naturally harbor an almost complete metabolic route for HA synthesis, just lacking the HAS gene (hasA), such as *B. subtilis*, *E. coli*, and *C. glutamicum* (Manfrao-Netto et al., 2021). Based on the heterologous expression of *hasA*, different artificial *has* operons containing a combination of different genes have been constructed in various hosts to increase HA titer. Examples are *sp*ABC and *sse*AB in *E. coli* (Yu and Stephanopoulos 2008), *pmhasA*-*tuaD*-*gtaB* (operon *has*ABC) in *B. subtilis* 168 (Jia et al., 2013), and *ssehasA-hasB* operon in *C. glutamicum* (Cheng et al., 2016), which in general obtained about 1–7 g/L of HA. Besides, engineering metabolic pathways promoted the yield of HA as well in different strains (Jin et al., 2016; Westbrook et al., 2018a). Wang et al. (2020) coupled HA degradation with HA production through adding leech hyaluronidase, which led to 74.1 g/L of HA accumulation with super-low M_W_ (∼53 kDa). In addition, some researchers also found that the cell-morphology engineering strategies, for example, downregulating or overexpressing the cell division initiator protein FtsZ in *B. subtilis* or *C. glutamicum*, can further enhance the HA titer (Westbrook et al., 2016; Zheng et al., 2020). For the overexpression of *Vitreoscilla* sp. hemoglobin (VHb), however, contrary results were reported in different strains: in *B. subtilis*, expression of VHb improved the HA titer from 0.9 to 1.8 g/L (Chien and Lee 2007); but in *C. glutamicum*, co-expression of the VHb gene (*vgb*) with *hasA* lowered HA yield by about 1.5-fold (Hoffmann and Altenbuchner 2014). In view of this, the effect of VHb varies case by case, which needs to be further investigated. Except for the genetic strategies, some researchers also enhanced the HA yield through optimization of the culture medium. Important functions of trace element and different kinds of carbon and nitrogen sources such as corn syrup powder and glucose were highlighted (Cheng et al., 2016; Chahuki et al., 2019).

Herein, we proposed four indirect pathway metabolic strategies to further enhance the HA titer in engineered *C. glutamicum*, i.e., enhancing the carbon substrate uptake via genetically activating the PTS-independent uptake system; regulating HA synthesis through the intensified synthesis of CL (an auxiliary molecule of HAS); duly expressing VHb via integration of the gene *vgb* into the genome of *C. glutamicum*, thereby promoting cell oxygen transfer, energy metabolism, and finally HA synthesis; and lastly finding the key inorganic nitrogen source (glutamine) through transcriptome analyses and then enhancing the HA titer via its supplementation. Combination strategies were further evaluated, and HA titer was significantly enhanced by optimal combinations.

## Materials and Methods

### Kits, Strains, Media, Plasmids, and Growth Conditions

DNA amplification was performed using 2× Phanta Max Master Mix, purchased from Vazyme (Nanjing, China). A DNA Gel Extraction Kit and Plasmid Miniprep Kit were purchased from Omega (Norcross, GA, United States). Gibson Assembly Reaction Kits were used, purchased from Clonesmarter Technologies (Scottsdale, AZ, United States). An ATP Assay Kit was obtained from Solarbio (Beijing, China), and a NAD^+^/NADH Assay Kit (WST-8 method) was purchased from Beyotime (Shanghai, China).

All the strains and plasmids used in this study were listed in Table 1, and the primers used for gene amplifications were listed in Supplementary Table S1. *E. coli* trans10 (TransGen Biotech Co., LTD) was used to construct and amplify the recombinant plasmids. Luria-Bertani broth (LB) medium (tryptone 10 g/L, yeast extract 5 g/L, and NaCl 10 g/L) was used for culturing *E. coli* strains. If necessary, 50 μg/ml kanamycin was added. *C. glutamicum* ATCC13032 was the starting host strain for subsequent *C. glutamicum* engineered strains. Cg-0 was constructed previously in our laboratory, and its genotype was shown in Table 1 (Cheng et al., 2019b). For flask culture, all of the engineered *C. glutamicum* strains were cultured in a 500 ml flask containing 50 ml fermentation medium (40 g/L glucose, 20 g/L (NH_4_)_2_SO_4_, 1 g/L K_2_HPO_3_, 0.5 g/L KH_2_PO_3_, 5 g/L MgSO_4_·7H_2_O, 0.01 g/L FeSO_4_·7H_2_O, and 0.01 g/L MnSO_4_, pH = 7.2) at 28°C, 200 rpm. The pH of the medium was adjusted to 7.2 every 12 h, and glucose was supplemented every 24 h.

**TABLE 1:** Plasmids and strains in this study.

Plasmids and strains	Description	Reference
Plasmids		
pEC-XK99E	Kana^r^, P_trc_-MCS, *rep* from native plasmid pGA1 (GenBank: X90817.2) of *C. glutamicum*	Yang et al. (2016)
pEC-AB	pEC-XK99E derivate, P_*tac*_-*hasA-hasB*	Cheng et al. (2019b)
AP_dapB_BaF	pEC-XK99E derivate, P_*tac*_-*hasA*-P_*dapB*_-*hasB*-Ter, P_tac_-as-F-Ter	Cheng et al. (2019b)
AP_dapB_B-*pgs*A1-aF	AP_*dapB*_BaF derivate, P_*dapB*_-*pgs*A1	This work
AP_dapB_B-*pgs*A2-aF	AP_*dapB*_BaF derivate, P_*dapB*_-*pgs*A2	This work
AP_dapB_B-*cls-*aF	AP_*dapB*_BaF derivate, P_*dapB*_-*cls*	This work
pK18mobsacB	Kana^r^, *sacB* from *B. subtilis*	Cheng et al. (2019a)
pK18mobsacB-Δ*iolR*	pK18mobsacB derivate, harboring upstream and downstream homologous arms of *iolR*	This work
pK18mobsacB-Δ*ldh*::*vgb*	pK18mobsacB derivate, harboring upstream and downstream homologous arms of *ldh*, and *vgb* gene	This work
Strains		
*E. coli* Trans10	F-*mcr*A Δ(*mrr*-*hsd*RMS-*mcr*BC)	TransGen
	φ80 *lac*ZΔM15Δ*lac*X74 *rec*A1 *ara*Δ139 Δ(*ara-leu*) 7697 *gal*U *gal*K *rps*L (Str^R^) *end*A1 *nup*G	
*C. glutamicum* ATCC13032	Wild type	Cheng et al. (2016)
Cg-ΔLACPZ	Wild-type derivate, Δ*ldh*, Δ*ackA-pta*, Δ*cat*, Δ*poxB*, Δ*zwf*	Cheng et al. (2019b)
Cg-0-half	Wild-type derivate, containing the plasmid pEC-AB, with half HA titer of Cg-0	Cheng et al. (2019b)
Cg-0	Wild-type derivate, Δ*ldh*, Δ*ackA-pta*, Δ*cat*, Δ*poxB*, Δ*zwf,* containing the plasmid AP_*dapB*_BaF	Cheng et al. (2019b)
Cg-dR	Cg-0 derivate, containing the plasmid AP_*dapB*_BaF, Δ*iolR*	This work
Cg-VHb	Cg-0 derivate, containing the plasmid AP_*dapB*_BaF, Δ*ldh*::*vgb*	This work
Cg-pgsA1	Cg-0 derivate, containing the plasmid AP_*dapB*_B-*pgs*A1-aF	This work
Cg-pgsA2	Cg-0 derivate, containing the plasmid AP_*dapB*_B-*pgs*A2-aF	This work
Cg-CLS	Cg-0 derivate, containing the plasmid AP_*dapB*_B-*cls*-aF	This work
Cg-dR-VHb	Cg-0 derivate, containing the plasmid AP_*dapB*_BaF, Δ*iolR*, Δ*ldh*::*vgb*	This work
Cg-dR-CLS	Cg-0 derivate, containing the plasmid AP_*dapB*_B-*cls*-aF, Δ*iolR*	This work
Cg-VHb-CLS	Cg-0 derivate, containing the plasmid AP_*dapB*_B-*cls*-aF, Δ*ldh*::*vgb*	This work
Cg-dR VHb-VLS	Cg-0 derivate, containing the plasmid AP_*dapB*_B-*cls*-aF, Δ*iolR*, Δ*ldh*::*vgb*	This work

Plasmid pK18mobsacB containing the *sacB* gene was used to conduct genome editing (such as *iolR* gene deletion) via double-crossover homologous recombination driven by sucrose selection (Schafer et al., 1994). Gene *vgb* was cloned from a plasmid in a previous study (Wang et al., 2018). The recombinant plasmid pK18mobsacB-Δ*ldh*::*vgb* was constructed and used for *vgb* integration into the genome. The genes relating to synthesis of CL (*pgsA1*, *pgsA2*, and *cls*) were amplified from the *C. glutamicum* genome and were fused into the plasmid AP_*dapB*_BaF, resulting in the plasmid AP_*dapB*_B-*pgs*A1/*pgs*A2/*cls*-aF. Competent cells of Cg-ΔLACPZ were used for construction of strain Cg-dR and Cg-VHb by homologous recombination via pK18mobsacB. Engineered strains Cg-dR, Cg-VHb, Cg-pgsA1, Cg-pgsA2, Cg-CLS, Cg-dR-VHb, Cg-dR-CLS, Cg-VHb-CLS and Cg-dR-VHb-CLS were constructed and utilized for studies in this work.

### Cell Growth Measurement (OD_600_) and HA Titer Assay

During flask cultivation, 1.5 ml broth sample was withdrawn at 24, 48, and 72 h, to determine the OD_600_ and HA titer of different recombinant strains. The HA titer was determined as follows: 3 ml ethanol was added into 1 ml fermentation broth, stored at 4°C for 2 h. The precipitation was collected by centrifugation (10,000 rpm, 3 min). Then 1 ml distilled water was added, and the HA yield was measured by the modified CTAB method, as previously described.

### Transcriptome Analysis of *C. glutamicum*

Cells of the two recombinant *C. glutamicum* strains (Cg-0 and Cg-0-half) cultured for 24 h were collected and centrifuged at room temperature for 10 min at 10,000 rpm and then stored at −70°C. The frozen cells were sent to Beijing Novogene to determine the transcriptome data.

### VHb Expression Verification Through Matrix-Assisted Laser Desorption/Ionization Time-of-Flight Mass Spectrometry

The broth samples of Cg-0 and Cg-VHb were collected at 24 h in parallel culture. After centrifugation at 13,000 rpm for 5 min, cell pellets were harvested and washed twice with phosphate buffer solution (PBS, 20 mM, pH = 7.2). Then the cell suspensions in PBS were sent for MALDI-TOF-MS analyses, using an ABI 4800 Plus analyzer (Applied Biosystems, Foster City, CA, United States).

### ATP and NAD^+^/NADH Measurement

ATP and NAD^+^/NADH results of Cg-0 or Cg-VHb were measured by an ATP Assay Kit (Solarbio) and NAD^+^/NADH Assay Kit (Beyotime), respectively. For both assays, 1 ml broth sample at 24 or 48 h with a cell concentration of 1 OD was withdrawn and centrifuged at 13,000 rpm for 5 min. The pellet was washed twice and resuspended with 1 ml PBS (pH = 7.2, 20 mM) and then used for ATP assay and NAD^+^/NADH measurement with the standard protocol of the kit.

### Fed-Batch Culture in a 10 L Fermenter

The fed-batch culture was conducted in a 10 L fermenter (Sartorius), containing 4 L fermentation medium as described above supplemented with 2 g/L glutamine. The aeration was set as 1 vvm, and agitation was set as 600 rpm, at 28°C. After 48 h of fermentation, the agitation was set as 800 rpm. The pH of the medium was retained at 7.2 by 8 M NaOH and 6 M HCl. The concentration of glucose was measured every 2 h after 8 h of fermentation. And 800 g/L glucose was added when its concentration dropped below 10 g/L, ensuring that the residual glucose concentration remained between 8 and 15 g/L.

## Results

**FIGURE 1:**
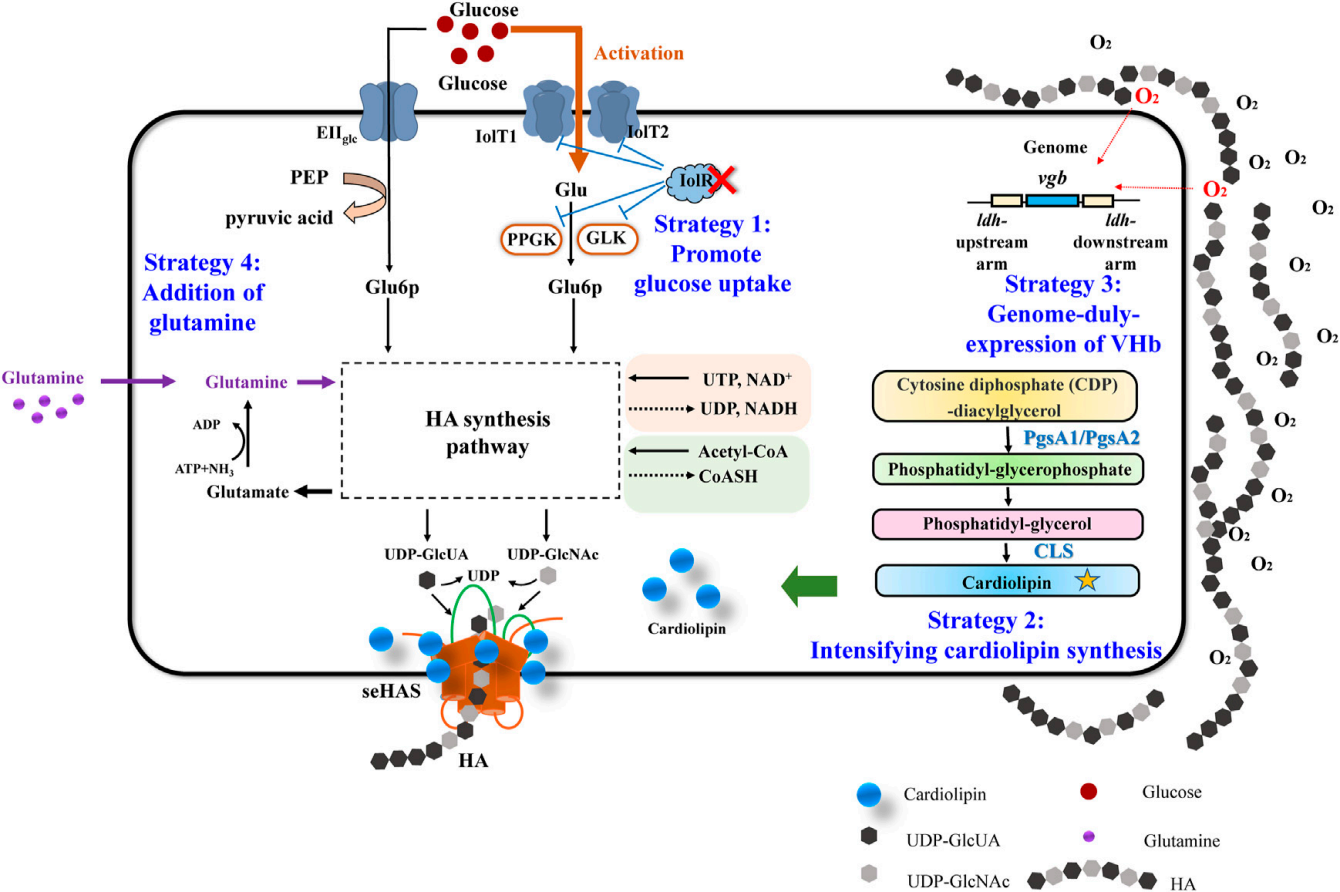
Overview of the four indirect metabolic strategies to promote HA production in this work.

As mentioned above, various efforts have been made to promote HA production in recombinant *C. glutamicum*, for example, optimization of *has* operon, knocking out competitive metabolic pathways, membrane engineering to enlarge the availability of cell membrane, and coupling HA synthesis with HA hydrolysis. Besides these, here, we tried to find out some strategies, indirect, non-regular, but effective as well, to further improve the HA titer in engineered *C. glutamicum*. Figure 1 showed the overview of the indirect metabolic strategies to promote HA production in this work.

### Enhancing HA Synthesis via Improvement of Glucose Uptake by IolR Deletion

As illustrated in Figure 1, Strategy 1 highlighted the promotion of glucose uptake via transport regulation. Carbon sources are essential factors for cell growth and target product synthesis. For most industrial fermentation processes, glucose is always the main carbon source due to its low price and high utilization efficiency. There are two glucose transport systems in *C. glutamicum*: phosphoenolpyruvate-dependent glucose phosphorylation via the phosphotransferase system (PTS^Glc^) and PTS-independent glucose uptake system (non-PTS^Glc^), such as the coupling system of myo-inositol permease and glucokinase (IPGS), and the coupling system of beta-glucoside-PTS permease and glucokinase (GPGS) (Ruan et al., 2020). For the IPGS pathway, glucoses are firstly transported into cells by myo-inositol permeases (IolT1/IolT2) and then phosphorylated by glucokinases (Glk and PpgK). The transcription of *iolT1*/*iolT2*/*glk*/*ppkg*, however, is repressed by a GntT-type regulator IolR in *C. glutamicum* (Klaffl et al., 2013). Deletion of the regulator IolR can strongly activate the non-PTS system and enhance the glucose uptake rate (Zhang B. et al., 2019). Therefore, deleting the IolR gene, thereby improving the glucose uptake efficiency, was proposed to be the first strategy to enhance HA titer.

**FIGURE 2:**
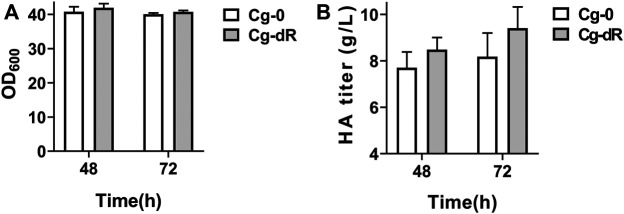
Cell growth and HA titer comparison of Cg-dR and control Cg-0. **(A)** OD_600_; **(B)** HA titer. Experiments were performed in triplicate.

As described in Table 1, the HA producer Cg-0 was utilized as the starting strain for subsequent studies, which could yield 6.2 g/L of HA in flask culture and 24.5 g/L of HA in fed-batch culture (Cheng et al., 2019b). The engineered Cg-dR was obtained after successful deletion of the gene *iolR* (Supplementary Figure S2). Effects of IolR deletion on cell growth and HA synthesis were evaluated via parallel flask culture of Cg-0 and Cg-dR. As shown in Figure 2, the OD_600_ of Cg-dR showed a litter difference with that of Cg-0, while the HA titer of Cg-dR was 6.9% higher than that of Cg-0 at 48 h, reaching 8.48 g/L.

### Enhancing HA Synthesis via Intensification of CL Synthesis—Auxiliary Factor of HAS

Strategy 2 focused on the auxiliary factor for maintaining the high activity of HAS, which is of great importance for HA biosynthesis. It was reported that CL was the activator for seHAS (Tlapak-Simmons et al., 1998; Tlapak-Simmons et al., 1999). The active *Streptococcal* HAS contains a single HAS monomer and multiple CL molecules (14–18 molecules of CL) (Tlapak-Simmons et al., 1998); and the exogenous CL could rescue the HAS activity (Kakizaki et al., 2002). CL, also known as diphospholipin, is synthesized from the intermediate metabolite cytosine diphosphate-diacylglycerol (CDP-DAG) by phosphatidylglycerophosphate synthase (PGS), phosphatidylglycerophosphate phosphatase (PTPMT), and CL synthase (CLS). Among them, the key enzyme phosphatidylglycerol phosphate synthase has two copies in the *C. glutamicum* genome, which are annotated as *pgsA1* and *pgsA2*, and the CLS is annotated as *cls*. In this study, the CL metabolic pathway was intensified via overexpression of *pgsA1*/*pgsA2*/*cls* genes, thereby indirectly enhancing the HAS activity and HA production.

**FIGURE 3:**
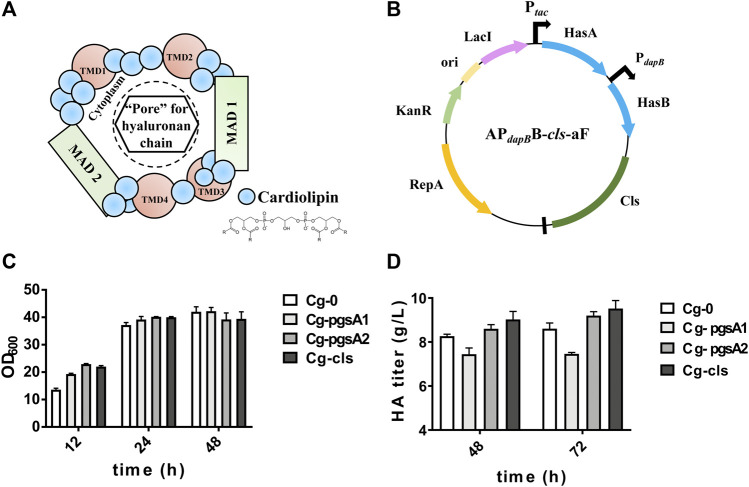
Cell growth and HA titer results after intensification of CL synthesis. **(A)** Hypothetical model of the active CLS–HAS complex: the scheme depicts the close association of CL molecules (blue dots) with one HAS protein to create an active enzyme. The growing HA chain would be transferred through this pore-like opening. The membrane domains of the HAS are labeled as transmembrane domains (TMDs) or membrane-associated domains (MADs) (Tlapak-Simmons et al., 1999). **(B)** Plasmid map of AP_*dapB*_B-*cls*-aF. **(C)** OD_600_ of the engineered strains Cg-pgsA1, Cg-pgsA2, and Cg-CLS. **(D)** HA titer of the same engineered strains. Strain Cg-0 was the control. Experiments were performed in triplicate.

The hypothetical “HAS–CL” complex model is shown in Figure 3A, in which CL molecules bind with different domains of HAS to form a “pore” for HA transportation. Three key enzymes for CL synthesis were figured out and labeled in Figure 1. Three engineered strains, Cg-pgsA1, Cg-pgsA2, and Cg-CLS overexpressing the above enzymes separately, were constructed and were evaluated in flask culture for 72 h (Figures 3C,D). For HA titer, *pgsA2* and *cls* both enhanced the HA synthesis by ∼10% (reaching 9.2 and 9.5 g/L, respectively) with respect to that of the control Cg-0 (8.6 g/L), although *pgsA1* reduced the HA titer by 14%.

### Enhancing HA Synthesis via the Duly Expressed VHb in Genome

HA synthesis is a high-energy-demand process, in which many reactions need UTP and NAD^+^. Besides, acetyl-CoA is also required. For example, 1 mol of glucose-1-P is transformed to UDP-glucose, consuming 1 mol of UTP; after that, 1 mol of UDP-glucose is converted into 1 mol of UDP-glucuronic acid, consuming 2 mol of NAD^+^ and releasing 2 mol of NADH. The metabolism or recycle of these co-factors and also cell growth all need oxygen. To meet the high-oxygen and high-energy requirements of HA production, increasing dissolved oxygen (DO) is regarded as an effective strategy for both native producers and recombinant strains. Traditional stirring or aeration-rate optimization usually causes high energy consumption and physical damage to the cells (Galaction et al., 2004). VHb, found in the obligate aerobic bacteria *Vitreoscilla*, can improve respiration and energy metabolism under oxygen-limited conditions (Orii and Webster 1986; Zhao et al., 2017). To duly express VHb via a genome-integrated way to enhance oxygen transfer and cell intake was proposed as Strategy 3 for enhanced HA production.

**FIGURE 4:**
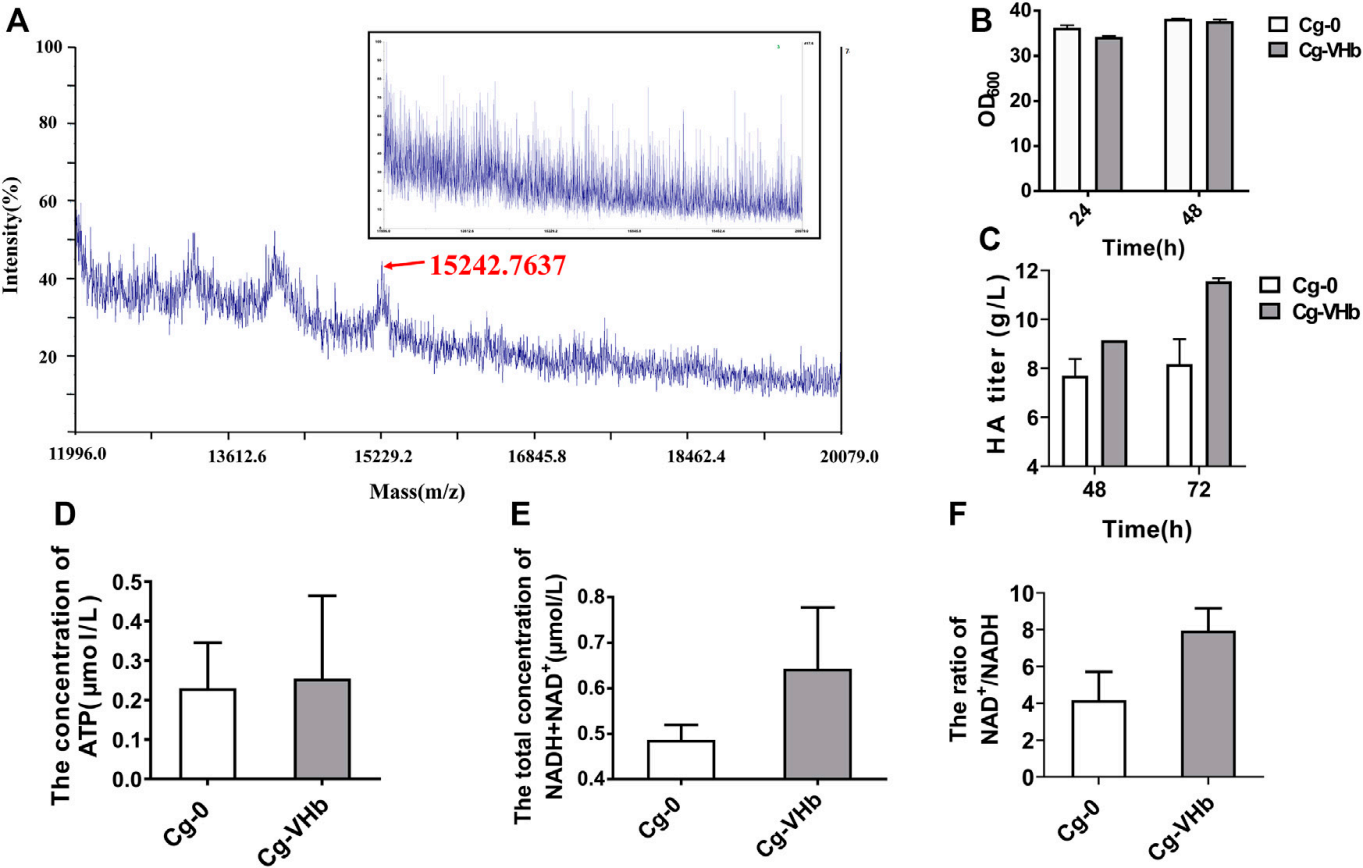
Enhancing HA synthesis via duly expressed VHb in genome. **(A)** MALDI-TOF-MS. **(B)** OD_600_. **(C)** HA titer. **(D)** ATP concentration. **(E)** Total concentration of NAD^+^ and NADH. **(F)** The ratio of NAD^+^ to NADH. In **(D)**, Cg-VHb and Cg-0 were cultured in a flask for 24 h, and in **(E**,**F)**, Cg-VHb and Cg-0 were cultured in a flask for 48 h. All of the measurements were performed in triplicate.

Engineered Cg-VHb was constructed by inserting the gene *vgb* into the site of the lactate dehydrogenase gene (*ldh*) (Supplementary Figure S3). MALDI-TOF-MS was adopted to verify expression of VHb, as shown in Figure 4A. In comparison with Cg-0, a new peak occurred in Cg-VHb at around 15 kDa, which was the expressed VHb. Flask culture results showed that although cell growth was not obviously changed (Figure 4B), the HA titer of Cg-VHb was highly increased by 26% at 72 h (11.56 g/L) compared with that of Cg-0 (Figure 4C).

The ATP content was further assayed for both Cg-0 and Cg-VHb. As shown in Figure 4D, intracellular ATP concentration of Cg-VHb was higher than that of Cg-0 by 10.7%. At the same time, the intracellular NAD^+^/NADH ratio was investigated as well. It could be seen that the total concentration of NAD^+^ and NADH in Cg-VHb was higher than that of Cg-0 by 32.3% and that Cg-VHb generated more NAD^+^, resulting in a higher NAD^+^/NADH (>7.5) ratio of Cg-VHb than that of Cg-0. These results indicated that introduction of VHb indeed intensified uptake of oxygen, especially in the late stage of fermentation, thereby leading to more ATP and promoting the energy metabolism.

### Combination of the Indirect Genetic Strategies

**FIGURE 5:**
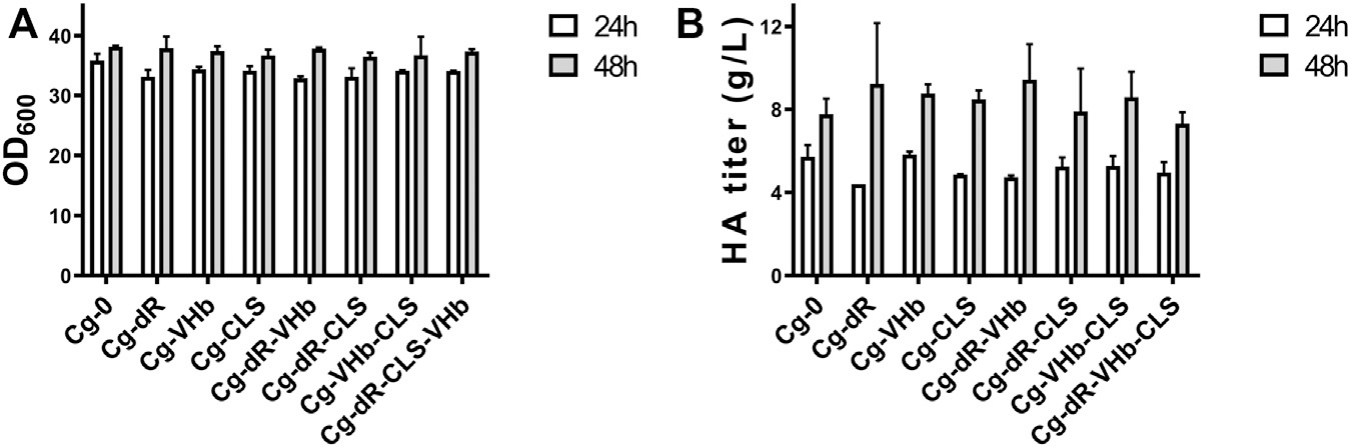
Combination of different indirect genetic strategies on cell growth and HA synthesis. **(A)** OD_600_ of different strains. **(B)** The HA titer of different strains. Strain Cg-0 was the control. Experiments were performed in triplicate.

With combination of the above genetic strategies, we further constructed four engineered strains, i.e., Cg-dR-VHb, Cg-dR-CLS, Cg-VHb-CLS, and Cg-dR-CLS-VHb. We assayed the cell concentrations (Figure 5A) and HA titers (Figure 5B) at 24 and 48 h in flask culture for different strains. At 48 h, Cg-dR-VHb showed the highest HA yield, reaching 9.43 g/L. But surprisingly, the triple-strategy strain Cg-dR-VHb-CLS behaved even worse than the control, which should be further investigated.

### Effect of Glutamine and Combination of Four Strategies on Cell Growth and HA Synthesis

According to the synthesis pathway of HA in recombinant *C. glutamicum* (Supplementary Figure S1), one molecule of glutamine is required together with UTP, NAD^+^, and acetyl-CoA (Figure 1). Glutamine provides an amino group for fructose 6-phosphate (F6P), which is converted into GlcN-6P by glutamine-fructose-6-phosphate aminotransferase, GlmS. Obviously, lots of glutamines will be consumed during HA synthesis. Thereby, we investigated the importance of glutamine for HA synthesis via both transcriptional analysis and glutamine supplementation, which was regarded as the fourth strategy.

To ensure the significance of glutamine on HA synthesis, transcriptome analysis was specifically performed with two controls, Cg-0 and Cg-0-half (the HA titer was only half of Cg-0). In total, 242 upregulated genes were identified. Not considering the putative protein genes, artificially expressed genes, and structural protein genes, five genes, *cgl2910*, *proB*, *cgl0453*, *hisH*, and *cgl0448*, were found to be related with glutamate/glutamine metabolism, as listed in Table 2.

**TABLE 2:** Identified key proteins with significant transcription difference in Cg-0 and Cg-0-half transcriptome.

Genes	Gene description	Readcount	Readcount
		Cg-0	Cg-0-half
*cgl2910*	Pyruvate kinase	1,426.7924	641.107
*proB*	Glutamate 5-kinase	934.6316	403.2274
	Catalyzing the formation of l-glutamate 5-semialdehyde from l-glutamate which is the precursor of l-proline		
*cgl0453*	Catalyzing the formation of 2,5-dioxopentanoate, which is the precursor of glutamate	146.2368	60.8088
*hisH*	Glutamine amidotransferase class I	516.5292	228.2846
*cgl0448*	Type 1 glutamine amidotransferase-like domain-containing protein	245.6064	170.6814

**FIGURE 6:**
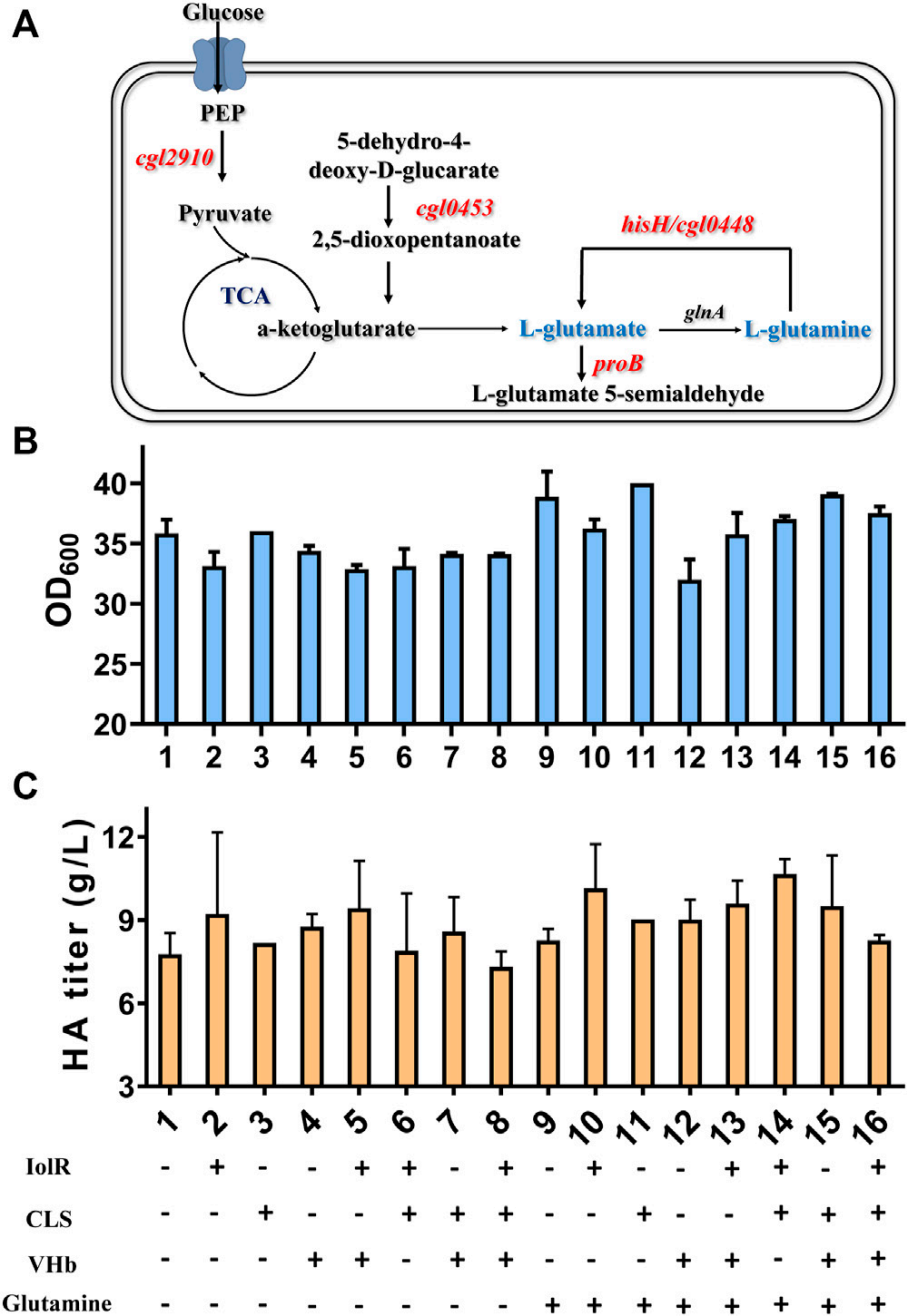
Glutamine metabolic pathway and effects of combined four strategies on cell growth and HA synthesis. **(A)** The key enzymes identified by transcriptome analysis are involved in glutamate or glutamine synthesis. **(B)** OD_600_ results of eight engineered strains under 16 different conditions. **(C)** HA titer of eight engineered strains under 16 different conditions.

**FIGURE 7:**
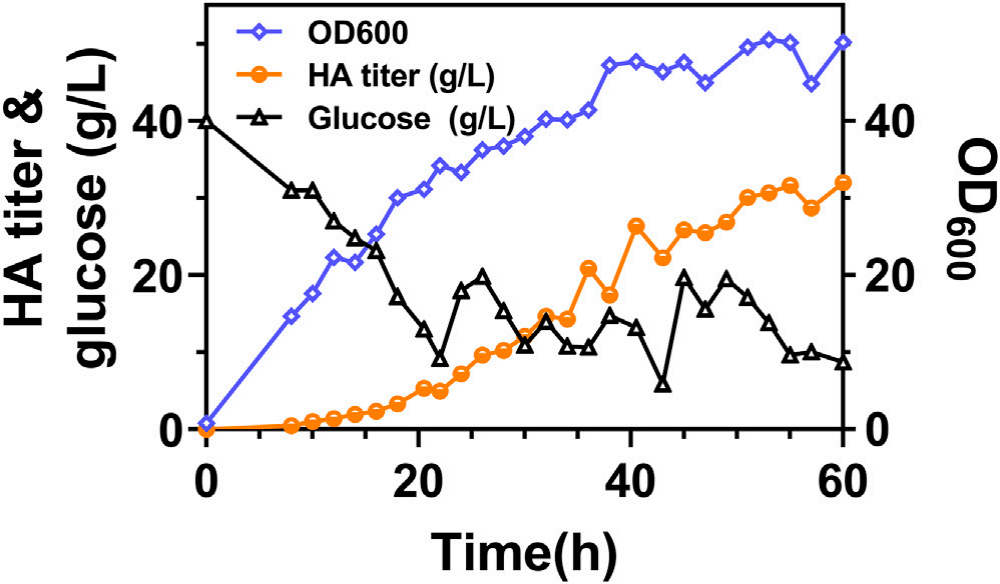
The time profiles of fed-batch fermentation by Cg-dR-CLS. Glucose feeding started at 22 h.

It can be found that the products of these five genes are just the enzymes involved in the metabolism of glutamic acid and glutamine, as shown in Figure 6A. So next, we added 2 g/L of glutamine into the medium and conducted flask culture for 48 h to test the cell growth and HA accumulation characteristics. As shown in Figures 6B,C, both OD_600_ and HA synthesis were significantly affected by glutamine addition, especially for Cg-dR-CLS and Cg-VHb-CLS. The highest HA titer, 10.66 g/L, was achieved by Cg-dR-CLS with glutamine, while the control Cg-0 only accumulated 8.26 g/L HA under the same conditions. Further, we performed fed-batch culture in a 10 L fermenter by Cg-dR-CLS, and the time profiles were shown in Figure 7. It can be seen that the HA titer of Cg-dR-CLS at 60 h reached 32 g/L, HA yield on glucose was 0.28 g/g, and HA productivity was 0.53 g/L/h. These results were higher than those of the superior strain CgHA25 we previously reported (Cheng et al., 2019b). This indicated that the strategies in this work performed well during the scale-up process.

## Discussion

In this study, we strengthened the biosynthesis of HA in recombinant *C. glutamicum* through four indirect, non-regular metabolic strategies. We assumed the HA-producing strain as a “whole block factory” and enhanced the product synthesis via intensified bioprocesses, such as substrate uptake, HAS activity improvement, and oxygen transfer.

Firstly, glucose uptake was intensified by IolR knockout, leading to a 6.9% increase of HA titer. This is a common effective strategy for enhancing the titer of different products synthetized by *C. glutamicum*. For example, deletion of IolR and overexpression of IolT1 also increased the yield of l-ornithine by 10% (Zhang B. et al., 2019). Similarly, Zhang X. et al. (2019) found enhanced cell growth and l-serine production of 3.9-fold and 5.9-fold, respectively, via inactivation of IolR. Based on the same metabolic regulation strategy, some other alternative strategies can also be tried to strengthen carbon utilization and thereby enhance the product yield, such as overexpression of the glucose transporters IolT1/T2 and EII_glc_.

CLs can bind HAS to form complexes and play an essential role in maintaining the biological activity of HAS. In a cell-free system, Weigel et al. reported that addition of CL into purified HAS increased the *K*_m_ for UDP-GlcUA and decreased the *K*_m_ for UDP-GlcNAc, finally giving an overall stimulation of V_max_. Both the seHAS and spHAS could maintain ∼60% initial activity by addition of bovine CL after being stored at −80°C for 2 months (Tlapak-Simmons et al., 1999). Based on the literature, we analyzed the CL synthesis pathway in *C. glutamicum* and focused on three key enzymes, PgsA1, PgsA2, and Cls.

We found that overexpression of *pgsA1/pgsA2/cls* can all accelerate cell growth to some degree, especially in the first 24 h. As reported previously, the *pgsA* gene codes for phosphatidylglycerophosphate synthase, which catalyzes the committed step of biosynthesis of phosphatidylglycerol (Dowhan 1997). And the *cls* gene codes for CLS, which condenses two molecules of phosphatidylglycerol to form CL in the prokaryote. As the major acidic phospholipids of the organism, phosphatidylglycerol and CL play important roles in bacterial cell structure and diverse physiological processes, such as DNA replication, cell division, respiration, and osmotic stress response (Wood 2018). Yasuhiro Shiba et al. (2004) reported that a *pgsA*-null mutation is lethal for *E. coli*. Xia et al. confirmed that *E. coli* are dependent on phosphatidylglycerol for cell growth, which cannot be substituted with phosphatidylinositol (Xia and Dowhan 1995). It can be seen that phosphatidylglycerol is essential for cell growth. As for CL, Kazuhisa Sekimizu et al. found that CL could activate the dnaA protein, which serves as the initiation of the protein of replication (Sekimizu and Kornberg 1988). Amer H. Asseri et al. (2021) reported that CL can also enhance the enzymatic activity of cytochrome *bd* for *C. glutamicum*, which is a terminal oxidase of respiratory pathways and can also enhance the oxygen consumption activity by twofold. Satomi Nishijima et al. (1988) came to the conclusion that the *cls* gene may confer growth or survival advantages for *E. coli*. In summary, *pgsA1/pgsA2/cls* genes are vital for cell growth and other physiological processes, and overexpression of these genes may promote cell growth to a certain degree.

In addition, we also found that *pgsA1* overexpression had a negative effect on HA titer, while *pgsA2* and *cls* could significantly promote the HA synthesis. We measured the concentration of free CLs in Cg-0 and Cg-CLS (Supplementary Table S2) and found that overexpression of *cls* led to an obvious increase of intracellular CLs. As for the negative effect of *pgsA1* on the HA titer, we assumed that *pgsA1* and *pgsA2* might have different activities towards conversion of CDP-DAG, thus leading to different concentrations of CL. In addition, Triscott and Vanderijn (1986) reported that the optimal activity of HAS occurred at a CL/protein ratio (μg/μg) of 5:1. In the future, we can clone *pgsA1*/*pgsA2*/*cls* and HAS under different inducible promoters and then accurately adjust the ratio of CL/HAS, thereby further enhancing the HA synthesis.

The HA synthesis process requires high-oxygen and high-energy conditions. It was observed that a higher HA titer was achieved in aerobic conditions than in anaerobic conditions (Huang et al., 2006; Prasad et al., 2010). According to our previous study, the recombinant *C. glutamicum* can produce HA with M_w_ ranging from 0.2 to 0.3 MDa (Cheng et al., 2019b). It was reported that when the M_w_ of HA was above 5.7 × 10^4^ Da, the broth viscosity increased with the HA concentration increasing, which would result in a great obstacle for nutrient and oxygen transfer. Nowadays, the industrialized HA titer was 6–10 g/L with a M_w_ of 2 MDa in *Streptococcus* (Cheng et al., 2019b). The high viscosity of fermentation media made the elevation of the HA titer in *Streptococcus* much more difficult. Therefore, to duly express VHb is probably an effective solution for this. Zhao et al. (2017) found that expression of VHb in *E. coli* resulted in a 94.4% increase of *trans*-4-hydroxy-l-proline production in a 100 ml shaking flask culture compared to the same strain without VHb expression. Wang et al. (2018) cloned and expressed VHb in a surfactin-producing strain *B. subtilis* THY-15, leading to a 24% increase in flask. But surprisingly, in *C. glutamicum*, co-expression of the VHb gene (*vgb*) with *hasA* based on plasmids lowered HA yield by 1.5-fold (Hoffmann and Altenbuchner 2014). Therefore, we deduced that duly expressing VHb is important for its positive function. We introduced VHb into the host via a genome-integrated strategy. After introduction of VHb, intracellular ATP and NAD^+^ increased dramatically, indicating that the energy metabolism was improved during fermentation. And it was assumed that more oxygen strengthened the oxidative phosphorylation process, thus improving NAD^+^ replenishment as well as the ratio of NAD^+^/NADH (Garrigues et al., 1997; Lan et al., 2006; Guez et al., 2008).

Besides, expression of the *vgb* gene increased the total concentration of NADH and NAD^+^. This could be attributed to the increased activity of the tricarboxylic acid (TCA) cycle (Pablos et al., 2014). It was reported that VHb can capture oxygen and transfer it to the terminal oxidases, and the dissociation rate constant of VHb is significantly higher than other hemoglobins. *E. coli*-expressing *vgb* would direct a higher fraction of glucose through the pentose phosphate pathway (ppp) and channel less acetyl-CoA through TCA than the wild-type strain, which generated an excess amount of NADPH, and resulted in a transhydrogenation reaction, leading to an H^+^-flux from NADPH to NAD^+^. To sum up, the effective delivery of oxygen to the cytochromes would regenerate NAD^+^ faster, activating the TCA cycle as well. During the dynamic balance of NADH and NAD^+^, the generation rate of NAD^+^ got faster, which probably led to the increase of the total concentration of NAD^+^ and NADH. As for the HA titer, we found that the HA titer of Cg-VHb was highly increased by 26%. Therefore, we supposed that integration of the VHb gene into the host genome is a promising strategy for industrialized HA bioproduction.

Finally, we investigated the influence of another important factor, amino group carrier (glutamine), on HA synthesis. We analyzed the transcriptome differences between HA high-producing strain and HA low-producing strain and identified that five genes relating to the metabolism of glutamate or glutamine were upregulated significantly. This indicated that when producing HA, the bacteria would enhance their native glutamic acid and glutamine synthesis to enhance the supplement of inorganic nitrogen for HA synthesis. To confirm this deduction, we tested the effectiveness of the exogenous addition of glutamine. Correspondingly, we found that the titer of HA was increased significantly. These results also implied that deficient supplement of glutamine will limit HA bioproduction to some degree. Aside from addition of glutamate/glutamine into the medium, we can also strengthen the synthesis of glutamine or glutamic acid in the engineered strains, for example, by overexpression of glutamine or glutamate synthase.

To conclude, four indirect metabolic engineering strategies for HA titer enhancement in engineered *C. glutamicum* were proposed and investigated in this work. In general, the enhancement of carbon uptake efficiency (specifically glucose), the auxiliary factor titer of HAS (CL), oxygen-transfer efficiency via duly expressing VHb, and supplement of glutamine all played positive and significant roles for enhanced HA synthesis. Combination strategies further elevated the HA titer, and the optimal strains showed a 30% increase in HA production under present conditions. It can be expected that after overall optimization and accurate regulation of different strategies next, the HA titer will be further increased significantly and thereby enable the scaled-up production of HA via the engineered *C. glutamicum*.

## Data Availability

The original contributions presented in the study are included in the article/Supplementary Material, further inquiries can be directed to the corresponding author.
