# Supplementary material for: Indirect Pathway Metabolic Engineering Strategies for Enhanced Biosynthesis of Hyaluronic Acid in Engineered Corynebacterium glutamicum
Source: Front Bioeng Biotechnol. 2021 Dec 20;9:768490. doi: 10.3389/fbioe.2021.768490 (PMC8721151; doi:10.3389/fbioe.2021.768490)
Supplement: Supplementary file 1 [file DataSheet1.docx]

Supplementary Material

# Supplementary Figures and Tables

## Supplementary Figures

**
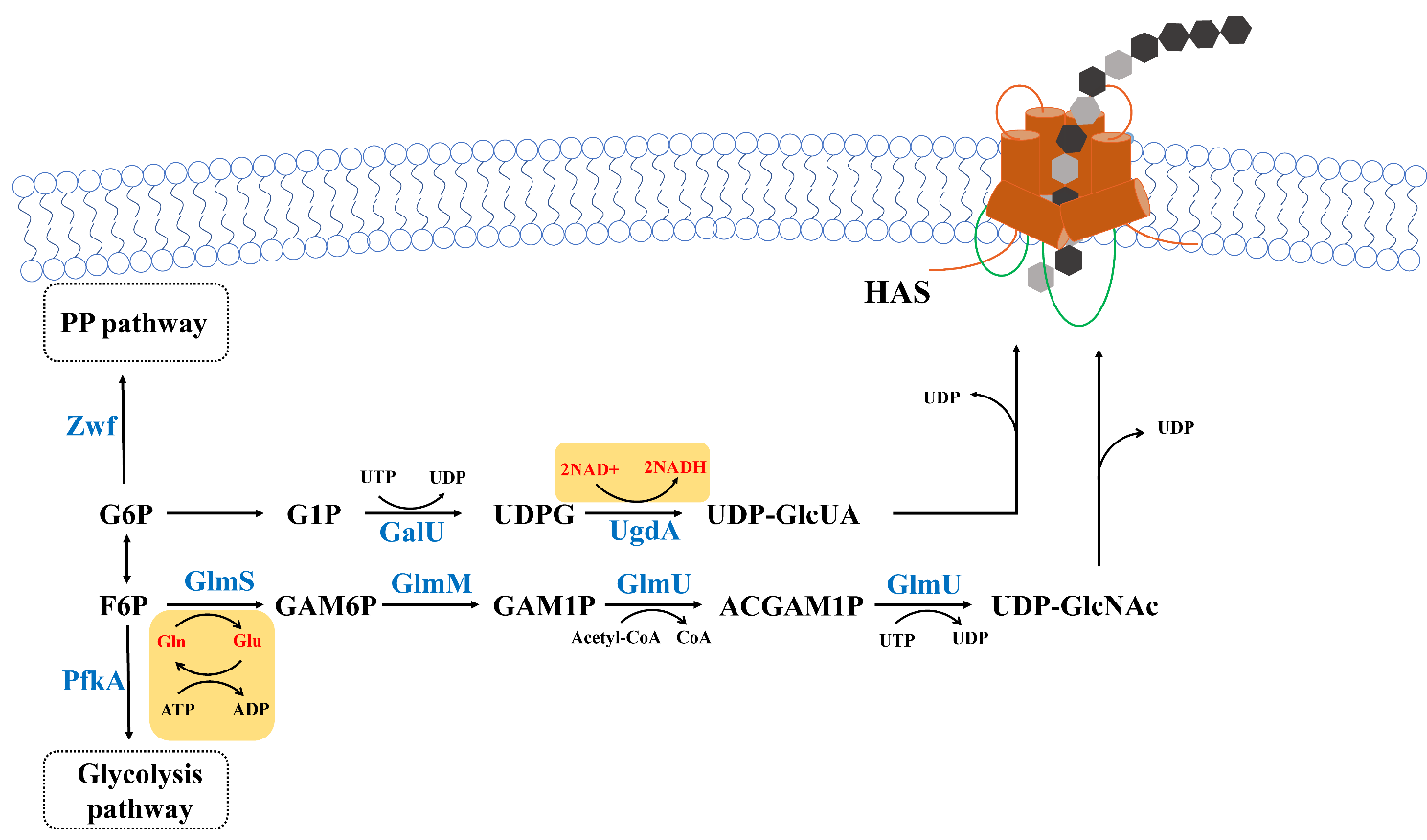
**

**Supplementary Figure S1. The HA synthesis pathway in recombinant *Corynebacterium glutamicum*.** Metabolites and enzymes are depicted in capital letters. PP pathway: pentose phosphate pathway; G6P: glucose-6-phosphate; G1P: glucose-1-phosphate; UDPG: UDP-glucose; UDP-GlcUA: UDP-glucuronate; F6P: fructose-6-phosphate; GAM6P: glucosamine-6-phosphate; GAM1P: glucosamine-1-phosphate; ACGAM1P: N-acetyl-glucosamine-1-phosphate; UDP-GlcNAc: UDP-N-acetyl-glucosamine; Zwf: glucose 6-phosphatedehydrogenase; PfkA: ATP-dependent 6-phosphofructokinase; GalU: UTP--glucose-1-phosphate uridylyltransferase; UgdA: UDP-glucose 6-dehydrogenase; GlmS: glutamine--fructose-6-phosphate aminotransferase; GlmM: phosphoglucosamine mutase; GlmU: bifunctional enzyme, includes: UDP-N-acetylglucosamine pyrophosphorylase, EC 2.7.7.23 (N-acetylglucosamine-1-phosphate uridyltransferase); glucosamine-1-phosphate N-acetyltransferase, EC 2.3.1.157; HAS: HA synthase.


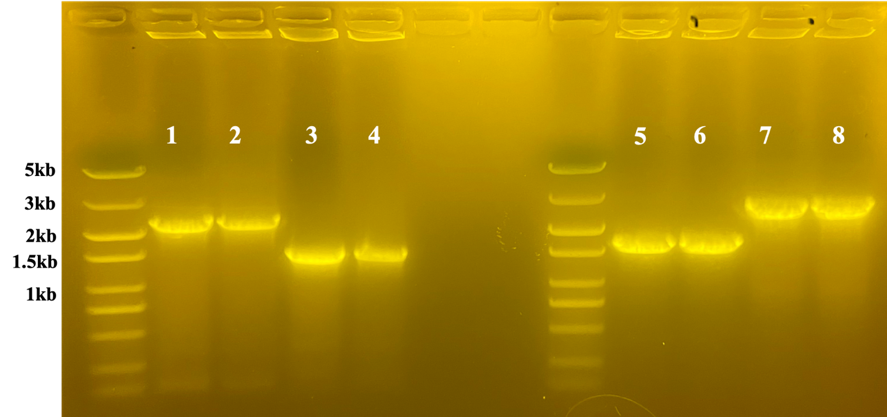


**Supplementary Figure S2.** **Determination of *iolR*** **deletion**. Lane 1-2: PCR of wild type *Corynebacterium glutamicum* ATCC13032 genome with primers d*iolR*-F1 and d*iolR*-F3; Lane 3-4: PCR of Cg-dR genome with primers d*iolR*-F1 and d*iolR*-F3.


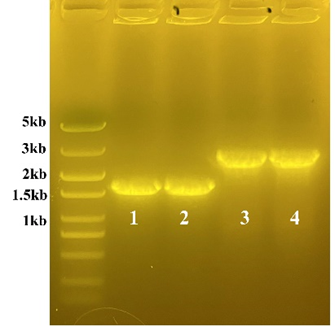


**Supplementary Figure S3.** **Determination of *vgb* insertion.** Lane 1-2: PCR of Cg-$\Delta LACPZ$genome with primers *vgb*-tests and *vgb*-testa; Lane 3-4: PCR of Cg-VHbgenome with primers *vgb*-tests and *vgb*-testa.

**Intracellular free cardiolipins in Cg-0 and Cg-CLS**

The intracellular free cardiolipins were assayed using Enzyme-liked Immunosorbent Assay Kit for Cardiolipin (CL) (Cloud-Clone Corp., CCC, USA), and the assay was conducted per the instruction manual. The standard curve was as follows:(Create a standard curve with the log of cardiolipin concentration on the Y-axis and absorbance on the X-axis).


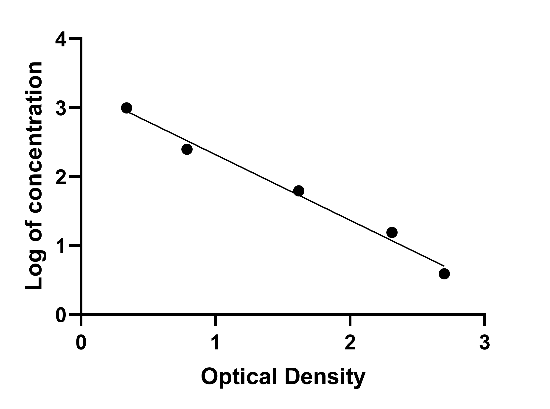


**Supplementary Figure S4 Standard curve for cardiolipin assay**


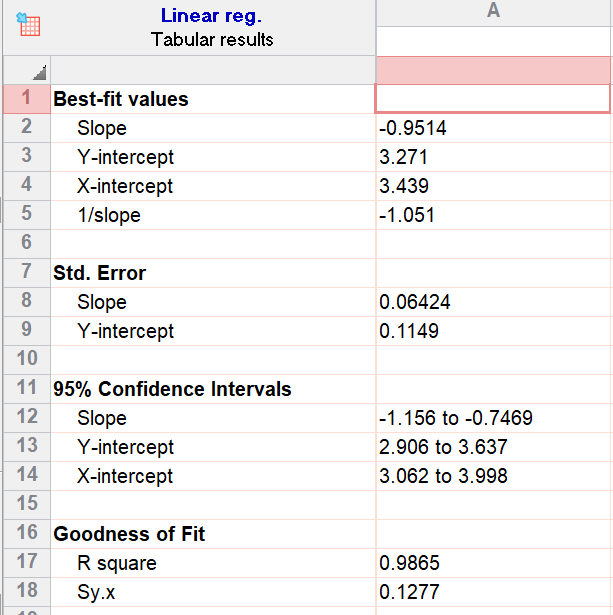


**Supplementary Figure S5 Linear fit analysis of the cardiolipin assay standard curve**

**
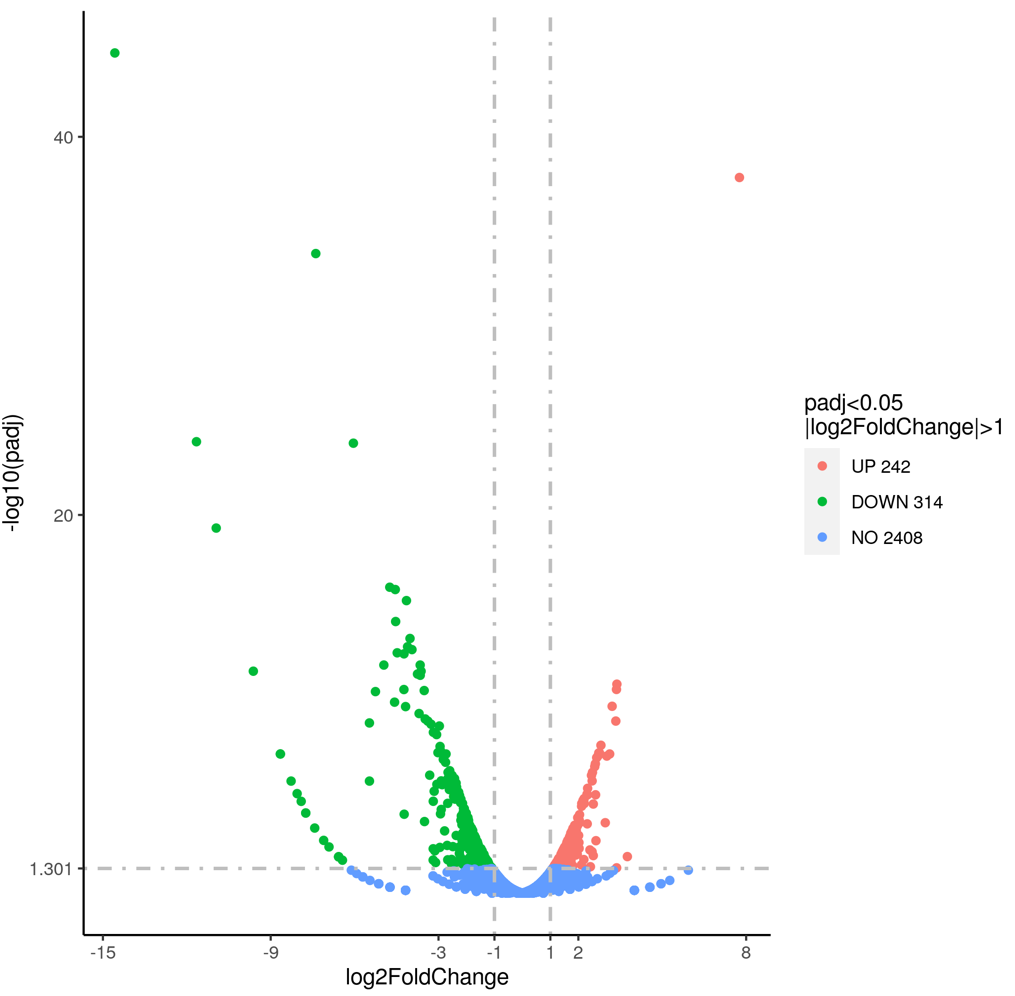
**

**Supplementary Figure S6 Volcano plot of transcriptome differences in Cg-0-half and Cg-0**

## Supplementary Tables

**Supplementary Table S1 Primers used in this study**

| **Primer name** | **Primer sequence (5’-3’)** |
| --- | --- |
| *iolR*-up-F | ACCATGATTACGAATTCGGTCTGGCTATCTACATCC |
| *iolR*-up-R | AACGAAAGGAGACATCGAAATAAACCAAAGAGCCC |
| *iolR*-down-F | TCGATGTCTCCTTTCGTTGCCCACC |
| *iolR*-down-F | TGCAGGTCGACTCTAGACAGAAACGTTTGCTGCGC |
| d*iolR*-F1 | ATCGGTTGGACTCTCATCGCC |
| d*iolR*-F2 | GGATGTGCTGCAAGGCGATTAAG |
| d*iolR*-F3 | GCAACGTACTGGTTGTCCACG |
| *ldh*-up-F | CTATGACCATGATTACGAATTCAGGTGCCGACACTAATGC |
| *ldh*-up-R | ACCGACGGTTTCTTTCATTTTCGATC |
| *ldh*-down-F | CTTCAGGTGTGCCTTGGCATAACTTTTTGGTTTACGGGCACAATG |
| *ldh*-down-R | TGCCTGCAGGTCGACTCTAGAGCTTCCAGACGGTTTCATCG |
| *vgb*-F | GAAAATGAAAGAAACCGTCGGTAAGCTTACAGGACGCTGGGG |
| *vgb*-R | ATGCCAAGGCACACCTGAAG |
| *vgb*-tests | TTCATCGATGTCGATGATCGC |
| *vgb*-testa | ACCACGAGATGGAACGCTTCAAG |
| SacI-*pgsA1*-F | ATAGAGCTCAAAGGACACATATGCAGGTAGGGTGGAACACCG |
| SacI-*pgsA1*-R | CCAATAGAGCTCCTAGCTTTCTGCGGACTTTCGTGAA |
| SacI-*pgsA2*-F | ATAGAGCTCAAAGGACACATATGCTGGGACTTCATGGACGTAAGC |
| SacI-*pgsA2*-R | CCCGAGCTCTTATTTGGTGTTGCTGTAATCTGCC |
| SacI-*cls*-F | CCGGAGCTCATGATCTTTCAGATCAACCTCGAAT |
| SacI-*cls*-R | ATAGAGCTCCTACTGCAGCGCCGAGGTCAAACGC |

**Supplementary Table S2 The intracellular concentration of cardiolipin**

| Strains | Cardiolipin concentration (ng/mL) |
| --- | --- |
| Cg-0 | 15.82 |
| Cg-CLS | 30.56 |

**Supplementary Table S3 All of the up-regulated genes**

| **Gene ID** | **Cg-0**  **Readcount** | **Cg-0-half**  **Readcount** |
| --- | --- | --- |
| CGL_RS06455 | 15.93132386 | 1.072442103 |
| CGL_RS13545 | 11764.98202 | 1131.800267 |
| CGL_RS06465 | 12.18151344 | 1.072442103 |
| CGL_RS06855 | 4183.830471 | 406.4276138 |
| sRNA00021 | 454.657612 | 44.80799952 |
| CGL_RS09820 | 3451.682658 | 373.3591545 |
| CGL_RS07520 | 260.6056277 | 29.87385422 |
| CGL_RS00360 | 320.6023769 | 39.47437618 |
| CGL_RS07315 | 59.05403209 | 7.472659693 |
| CGL_RS07820 | 6175.909988 | 882.1867365 |
| CGL_RS03000 | 4658.179758 | 701.9102979 |
| CGL_RS07815 | 3321.377221 | 510.9666141 |
| CGL_RS03005 | 11182.82608 | 1773.968408 |
| CGL_RS12725 | 46.86718686 | 7.472659693 |
| CGL_RS12610 | 190.2969363 | 30.94057886 |
| CGL_RS01295 | 4436.004302 | 727.5116857 |
| CGL_RS07810 | 3325.127018 | 553.6355938 |
| CGL_RS04700 | 161.2360099 | 27.74040498 |
| CGL_RS10005 | 25.30583556 | 4.272519241 |
| CGL_RS07830 | 8191.425734 | 1443.283817 |
| CGL_RS06155 | 36.55523856 | 6.405942471 |
| CGL_RS07825 | 10521.92441 | 1899.841898 |
| CGL_RS00600 | 17.80622741 | 3.205818546 |
| CGL_RS08990 | 40.30503828 | 7.472659693 |
| CGL_RS06205 | 3400.122953 | 675.2421857 |
| CGL_RS05310 | 122.80059 | 24.54023125 |
| CGL_RS04945 | 1052.750194 | 212.2837549 |
| CGL_RS03145 | 760.2660479 | 164.2811516 |
| CGL_RS13935 | 3435.746022 | 744.5792775 |
| CGL_RS15290 | 34.68033851 | 7.472659693 |
| CGL_RS09655 | 3630.735452 | 796.8487774 |
| CGL_RS13945 | 10876.2802 | 2422.536896 |
| CGL_RS13950 | 37405.15466 | 8595.671502 |
| CGL_RS05690 | 2279.871177 | 525.900757 |
| CGL_RS07435 | 23.43093419 | 5.339228359 |
| CGL_RS07270 | 316.8525801 | 76.80973846 |
| CGL_RS12250 | 923.3822064 | 224.0177245 |
| CGL_RS14670 | 65.61617876 | 16.0064369 |
| CGL_RS03340 | 95.61455987 | 23.47350673 |
| CGL_RS14140 | 137.7997785 | 34.14075283 |
| CGL_RS13700 | 1038.688456 | 259.2196331 |
| CGL_RS01205 | 408.7226015 | 103.4778529 |
| CGL_RS05305 | 404.0353555 | 103.4778529 |
| CGL_RS06220 | 54.36678418 | 13.8729896 |
| sRNA00034 | 83.42771809 | 21.34005782 |
| CGL_RS12615 | 159.3611114 | 41.60782552 |
| CGL_RS06550 | 320.6023769 | 84.2768106 |
| CGL_RS04820 | 56.24168338 | 14.93971313 |
| CGL_RS09150 | 74.99067339 | 20.27333344 |
| CGL_RS12850 | 313.1027833 | 85.34353518 |
| CGL_RS09695 | 1866.456087 | 510.9666141 |
| CGL_RS00925 | 854.9484158 | 234.6849695 |
| CGL_RS03150 | 711.5186901 | 198.4163362 |
| CGL_RS06820 | 104.9890532 | 29.87385422 |
| CGL_RS07055 | 5719.372235 | 1635.294225 |
| CGL_RS01535 | 242.7940927 | 69.34266622 |
| CGL_RS07625 | 2350.179866 | 673.1087367 |
| CGL_RS02995 | 37.49268853 | 10.6728212 |
| CGL_RS06825 | 85.30261686 | 24.54023125 |
| CGL_RS04100 | 195.9216317 | 56.54197071 |
| CGL_RS11255 | 224.9825576 | 65.07576776 |
| CGL_RS03055 | 1003.065387 | 291.2213682 |
| CGL_RS07630 | 3632.610351 | 1067.796797 |
| CGL_RS07245 | 1409.918334 | 416.0281343 |
| CGL_RS09580 | 446.2205693 | 132.2794157 |
| CGL_RS02310 | 962.7550722 | 286.9544702 |
| CGL_RS11210 | 193.109284 | 57.60869535 |
| CGL_RS14260 | 334.6641149 | 100.2776792 |
| CGL_RS12045 | 57.17913296 | 17.07316085 |
| CGL_RS13850 | 33887.84532 | 10222.42634 |
| CGL_RS10170 | 35.61778855 | 10.6728212 |
| CGL_RS14545 | 88.11496499 | 26.67368038 |
| CGL_RS07800 | 2016.447956 | 616.5723387 |
| CGL_RS01110 | 460.2823072 | 140.813212 |
| CGL_RS12855 | 1906.766402 | 584.570604 |
| CGL_RS13845 | 2667.037691 | 820.3167162 |
| CGL_RS01300 | 48.74208628 | 14.93971313 |
| CGL_RS14555 | 2811.404865 | 874.7196651 |
| CGL_RS14550 | 209.0459208 | 65.07576776 |
| CGL_RS07340 | 235.294499 | 73.60956465 |
| CGL_RS05210 | 91.86476246 | 28.80712959 |
| CGL_RS10820 | 159.3611114 | 50.14162283 |
| CGL_RS14950 | 225.9200068 | 71.47611544 |
| CGL_RS07220 | 1138.05807 | 360.5584606 |
| CGL_RS08000 | 1510.225397 | 478.9648794 |
| CGL_RS00630 | 949.6307836 | 302.9553377 |
| CGL_RS13345 | 548.4025312 | 176.0151214 |
| CGL_RS01070 | 195.9216317 | 62.94231851 |
| CGL_RS09660 | 576.5260069 | 185.615642 |
| CGL_RS13940 | 6865.872587 | 2229.459764 |
| CGL_RS00085 | 68.42852722 | 22.40678225 |
| CGL_RS12685 | 995.5657937 | 327.4900012 |
| CGL_RS11150 | 4248.514465 | 1408.081909 |
| CGL_RS07310 | 58.11658253 | 19.20660915 |
| CGL_RS07805 | 2051.133576 | 684.8427061 |
| CGL_RS12160 | 1037.751007 | 348.8244911 |
| CGL_RS12905 | 161.2360099 | 54.40852143 |
| CGL_RS11335 | 342.1637085 | 116.2785476 |
| CGL_RS15310 | 1185.867978 | 407.4943383 |
| CGL_RS11250 | 468.7193499 | 161.0809781 |
| CGL_RS13200 | 142.4870248 | 49.07489817 |
| CGL_RS10210 | 525.9037506 | 181.348744 |
| CGL_RS02860 | 49.67953596 | 17.07316085 |
| CGL_RS04110 | 2274.246482 | 785.1148081 |
| CGL_RS14250 | 487.4683338 | 168.5480497 |
| CGL_RS14150 | 390.9110668 | 135.4795893 |
| CGL_RS10525 | 647.7721453 | 225.084449 |
| CGL_RS03450 | 132.1750828 | 45.87472419 |
| CGL_RS06150 | 89.05241436 | 30.94057886 |
| CGL_RS13870 | 348.7258529 | 121.6121703 |
| CGL_RS08585 | 79.67792049 | 27.74040498 |
| CGL_RS05145 | 249.3562372 | 87.47698435 |
| CGL_RS08005 | 905.5706718 | 317.8894807 |
| CGL_RS11955 | 10521.92441 | 3705.806454 |
| CGL_RS04000 | 936.506495 | 330.6901747 |
| CGL_RS09965 | 4113.521782 | 1467.81848 |
| CGL_RS10815 | 1465.227836 | 525.900757 |
| CGL_RS05760 | 8387.352613 | 3030.569854 |
| CGL_RS03225 | 216.5455146 | 78.94318765 |
| CGL_RS01945 | 464.9695531 | 169.6147742 |
| CGL_RS12755 | 657.1466372 | 240.0185921 |
| CGL_RS04260 | 230.6072529 | 84.2768106 |
| CGL_RS01930 | 3688.857302 | 1352.612236 |
| CGL_RS07250 | 3484.493379 | 1281.141695 |
| CGL_RS03335 | 148.1117204 | 54.40852143 |
| CGL_RS05745 | 211.8582685 | 77.87646305 |
| CGL_RS06930 | 156.5487636 | 57.60869535 |
| CGL_RS09810 | 407.7851523 | 150.4137328 |
| CGL_RS10250 | 653.3968404 | 241.0853166 |
| CGL_RS06185 | 269.9801198 | 100.2776792 |
| CGL_RS05550 | 134.9874306 | 50.14162283 |
| CGL_RS14575 | 89.05241436 | 33.07402817 |
| CGL_RS04995 | 2060.508068 | 766.9804918 |
| CGL_RS06405 | 757.4537003 | 282.6875722 |
| CGL_RS06230 | 6494.64271 | 2424.670345 |
| CGL_RS00710 | 122.80059 | 45.87472419 |
| CGL_RS12810 | 1153.994706 | 435.2291752 |
| CGL_RS07285 | 2396.114876 | 906.7213998 |
| CGL_RS05675 | 165.9232561 | 62.94231851 |
| CGL_RS09025 | 455.5950612 | 173.8816723 |
| CGL_RS14145 | 958.0678263 | 365.892083 |
| CGL_RS07195 | 254.0434833 | 97.07750552 |
| CGL_RS02660 | 128.4252857 | 49.07489817 |
| CGL_RS05625 | 1912.391097 | 731.7785836 |
| CGL_RS11330 | 5228.148862 | 2007.581071 |
| CGL_RS06940 | 1320.860661 | 507.7664407 |
| CGL_RS15090 | 725.5804279 | 280.5541232 |
| CGL_RS00080 | 206.2335732 | 80.00991224 |
| CGL_RS09480 | 254.9809325 | 99.21095465 |
| CGL_RS08135 | 89.98986373 | 35.20747749 |
| CGL_RS07200 | 831.5121861 | 326.4232767 |
| CGL_RS03140 | 169.6730531 | 67.20921699 |
| CGL_RS14805 | 83.42771809 | 33.07402817 |
| CGL_RS09995 | 378.7242272 | 150.4137328 |
| CGL_RS15145 | 1086.498365 | 432.0290017 |
| CGL_RS12845 | 221.2327607 | 88.54370893 |
| CGL_RS10545 | 119.9882421 | 48.00817351 |
| CGL_RS15135 | 2244.248108 | 901.3877773 |
| CGL_RS09795 | 284.0418579 | 114.1450985 |
| CGL_RS03310 | 679.6454177 | 274.1537762 |
| CGL_RS06850 | 467.7819007 | 188.8158156 |
| CGL_RS10645 | 177.172647 | 71.47611544 |
| CGL_RS14825 | 7081.4859 | 2867.361008 |
| CGL_RS07240 | 168.7356039 | 68.27594161 |
| CGL_RS10085 | 235.294499 | 96.01078095 |
| CGL_RS10195 | 388.0987192 | 158.947529 |
| CGL_RS05630 | 348.7258529 | 142.9466611 |
| CGL_RS07280 | 4021.651762 | 1652.361817 |
| CGL_RS00640 | 236.2319482 | 97.07750552 |
| CGL_RS14600 | 254.0434833 | 104.5445775 |
| CGL_RS11950 | 11104.08035 | 4577.32036 |
| CGL_RS12365 | 411.5349491 | 169.6147742 |
| CGL_RS03825 | 915.8826129 | 377.6260525 |
| CGL_RS12125 | 228.7323545 | 94.94405638 |
| CGL_RS02305 | 146.2368218 | 60.80886926 |
| CGL_RS04425 | 1008.690082 | 421.3617568 |
| CGL_RS00280 | 241.8566435 | 101.3444038 |
| CGL_RS11155 | 432.1588314 | 181.348744 |
| CGL_RS04105 | 1402.41874 | 592.0376755 |
| CGL_RS09445 | 959.0052755 | 408.5610628 |
| CGL_RS00735 | 3616.673714 | 1543.555919 |
| CGL_RS00570 | 164.9858069 | 70.40939083 |
| CGL_RS03605 | 620.5861188 | 265.6199802 |
| CGL_RS09475 | 5115.65496 | 2195.324581 |
| CGL_RS07540 | 960.8801738 | 412.8279608 |
| CGL_RS05620 | 4508.187889 | 1940.377428 |
| CGL_RS14475 | 2375.490994 | 1022.994369 |
| CGL_RS06585 | 250.2936864 | 107.7447511 |
| CGL_RS11680 | 934.6315966 | 403.2274404 |
| CGL_RS10625 | 1113.684391 | 481.0983284 |
| CGL_RS09665 | 234.3570498 | 101.3444038 |
| CGL_RS10530 | 943.0686393 | 411.7612363 |
| CGL_RS15370 | 1677.091352 | 732.8453081 |
| CGL_RS12245 | 529.6535474 | 231.484796 |
| CGL_RS11520 | 1258.989015 | 550.4354203 |
| CGL_RS04930 | 7186.480208 | 3143.64265 |
| CGL_RS03795 | 243.7315419 | 106.6780266 |
| CGL_RS10405 | 516.5292587 | 228.2846225 |
| CGL_RS04760 | 255.9183817 | 113.0783739 |
| CGL_RS07965 | 1154.932155 | 512.0333386 |
| CGL_RS06180 | 869.9476027 | 386.1598485 |
| CGL_RS10175 | 1093.060509 | 485.3652263 |
| CGL_RS11345 | 654.3342896 | 291.2213682 |
| CGL_RS10955 | 771.5154381 | 343.4908686 |
| CGL_RS10190 | 5589.066798 | 2498.274335 |
| CGL_RS07445 | 2484.2351 | 1111.532501 |
| CGL_RS03900 | 6094.351909 | 2734.020447 |
| CGL_RS14485 | 1426.792419 | 641.107002 |
| CGL_RS14630 | 7206.166641 | 3249.248374 |
| CGL_RS09815 | 886.8216881 | 400.0272669 |
| CGL_RS08225 | 1376.170163 | 620.8392367 |
| CGL_RS12840 | 378.7242272 | 171.7482233 |
| CGL_RS01200 | 545.5901836 | 247.4856636 |
| CGL_RS05110 | 500.5926224 | 227.217898 |
| CGL_RS09610 | 605.5869318 | 275.2205007 |
| CGL_RS12120 | 393.7234144 | 179.2152949 |
| CGL_RS12875 | 2193.625852 | 1004.860053 |
| CGL_RS11020 | 1144.620214 | 528.034206 |
| CGL_RS14900 | 2935.148157 | 1357.945858 |
| CGL_RS11305 | 2917.336623 | 1359.012583 |
| CGL_RS07740 | 2533.919906 | 1180.869593 |
| CGL_RS07095 | 2015.510507 | 944.0567569 |
| CGL_RS04635 | 2156.127885 | 1011.2604 |
| CGL_RS15140 | 2457.986522 | 1156.33493 |
| CGL_RS04590 | 18856.7851 | 8878.353491 |
